# Sociodemographic characteristics and disability pensions of frequent attenders in occupational health primary care – a follow-up study in Finland

**DOI:** 10.1186/s12889-021-11873-8

**Published:** 2021-10-12

**Authors:** Tiia Reho, Salla Atkins, Mikko Korhonen, Anna Siukola, Markku Sumanen, Mervi Viljamaa, Jukka Uitti, Riitta Sauni

**Affiliations:** 1 Tampere University, Faculty of Medicine and Health Technology, Tampere, Finland; 2 Pihlajalinna Työterveys, Tampere, Finland; 3 Department of Public Health Sciences, Karolinska Institutet, Stockholm, Sweden; 4 Tampere University, New Social Research and Faculty of Social Sciences, Tampere, Finland; 5 Finnish Institute of Occupational Health, Tampere, Finland; 6 Clinic of Occupational Medicine, Tampere University Hospital, Tampere, Finland

**Keywords:** Frequent attender, Disability pension, Occupational health service, Health inequalities, Primary care, Healthcare utilization, Working age

## Abstract

**Background:**

Work disability is a complex issue that requires preventive efforts from healthcare systems and individuals, and that too often results in disability pensions (DP). While many studies have attempted to characterize risk factors of work disability, many showing for example a link between socioeconomic positions, working conditions and frequent attendance to OH primary care it is not known if frequent attendance is associated with DP despite the sociodemographic factors. This study aims to address this gap and examine the association between frequent attendance to OH primary care and DP, when adjusted by sociodemographic factors.

**Methods:**

This study combines routine medical record data of an occupational health service provider with comprehensive national registers. Medical record data were used to define groups of frequent attenders to OH primary care (FA) (1-year-FA, 2-year-FA, persistent-FA and non-FA) from 2014 to 2016. The sociodemographic factors (including i.e. educational level, occupational class, unemployment periods) were derived from Statistic Finland and DP decisions were derived from Finnish Centre for Pensions. Association of frequent attendance to OH primary care with DP decisions were analyzed and adjusted by sociodemographic factors.

**Results:**

In total, 66,381 patients were included. Basic and intermediate education along with manual and lower non-manual work predicted frequent attendance to OH primary care. Unemployment in 2013 did not predict frequent attendance to OH primary care. Frequent attendance to OH primary care was associated with DP within next two years, even when adjusted for sociodemographic factors. The association of frequent attendance to OH primary care with DP grew stronger as high service use persisted over time.

**Conclusions:**

Frequent attendance to OH primary care is associated with DP risk in the near future despite the underlying sociodemographic differences. Patients using OH primary care services extensively should be identified and rehabilitative needs and measures necessary to continue in the work force should be explored. Sociodemographic issues that co-exist should be explored and considered when planning interventions.

## Background

One tenth of patients conduct more than a third of primary healthcare visits and create an even larger proportion of costs [1–3]. This tenth is often termed frequent attenders to OH primary care (FAs), and they can be found in most healthcare systems creating a similar demand across settings [4–6]. The FA definition is not a stable one; some patients are FA only temporarily for a year, but others continue frequent visits for several years– the latter often being described as persistent FAs [1, 7, 8]. It is still unclear why some patients continue high service use while others cease and if these patients differ from each other.

FAs share some common characteristics including multimorbidity, worse self-perceived health and quality of life [3, 9–12]. In addition, unemployment has been linked to frequent attendance to OH primary care in the GP setting [13, 14] and low socioeconomic status has been linked to frequent service use and sickness absences [15]. Previous studies have established that frequent attendance is associated with a higher risk of work disability, also after frequent visits have ceased [16, 17]. However, as these studies have not controlled for sociodemographic factors, the question can be raised about the veracity of the frequent attendance to OH primary care as an independently associated variable. Sociodemographic factors, or the social determinants of health including low education levels and socioeconomic status, are established as risk factors for work disability [18–20]. Although the criteria for DP are medical, social determinants of health, including gender, education, income or poverty and employment act as background variables impacting on the occurrence and severity of disability [21]. Considering these factors and their relation to frequent attendance allows a more comprehensive understanding of work disability, and possibly, earlier measures to improve work ability.

Finnish occupational health services (OHS) is a unique environment to study the working population. The voluntary primary care provided by employers to employees covers up to 90% of the working population and it is often used as the sole primary care provider of the employees despite functioning parallel to private and public primary care [22, 23]. Occupational health (OH) primary care includes curative care, such as visits to physician and nurse, and through consultation also psychologist, physiotherapist and often medical specialists. However, the services are linked to employment relationship, and disruptions in employment will affect service use patterns. Although OH primary care as such does not exist elsewhere, in most countries the working population is treated by the GPs and understanding the characteristics of the working population FAs is necessary in these settings, too. There is minimal information on FAs among the working aged or the working population.

The working population is different from the general practice population as they have been fit enough to enter and stay in the work force [24]. However, the working population is heterogeneous in terms of illnesses and social and economic conditions [25, 26]. Frequent attendance exists in OH primary care similarly to other healthcare settings [4], but its predictors in OHS may be different when compared with the general population. The service demand and economic burden created by the FAs in OH primary care – a tenth makes over a third of the visits [4] – is notable but even more pressing is their association with work disability. Disability retirement is a loss for the workplace, the individual and the society both in economic and humane terms.

OHS in Finland aims to prevent work disability and withdrawal from the workforce in collaboration with employers and employees. To design purposeful interventions to those workers that are at risk of work disability – such as FAs – we need a wider conception of characteristics linked to FAs in OH primary care. With enough understanding on socioeconomic conditions that are linked to frequent attendance to OH primary care, we can identify the right groups for interventions but also plan the interventions purposefully. We also need further information on the independent risks associated with frequent attendance to OH primary care and work disability.

This study aimed to investigate the sociodemographic factors of different FA groups in OH primary care and investigate the association of frequent attendance to OH primary care with DPs.

## Methods

### Study setting and design

The Finnish OHS contain two structures: the mandatory preventive part and the voluntary primary care part, which are arranged by the same service provider. OH primary care are funded in most part by the employer, but when used it is free of charge for the employee. OH primary care functions alongside public and private primary care but is often used as the sole primary care provider [23]. This study is a retrospective follow-up study using routine medical record data from a large private OHS provider Pihlajalinna Työterveys, which at the time of the study included 40 OH units across Finland. A follow-up design was chosen to analyze factors associated with frequent attendance to OH primary care and DP. Pihlajalinna Työterveys’ clients represent the working population of Finland fairly well including companies from a wide range of industries and rural as well as urban areas.

While medical records contain the necessary information on patients’ illnesses and their treatment, they have little information on sociodemographic factors, such as occupational class or education. These are available from national registers controlled by Statistics Finland. This study combines routine medical record data that is used to identify FAs and combines it with sociodemographic data from Statistics Finland and further with disability pension data from Finnish Centre for Pensions (FCP).

In Finland, a DP may be granted when work ability is decreased for longer than a year based on an illness. When work ability is decreased by 2/5 a partial DP is granted and when it is decreased by 3/5 a full DP may be granted. Fixed-term solutions (partial or full-time) are granted when rehabilitation and return to work later is expected. Permanent full DP leads to withdrawal from the workforce. DPs are funded by a mandatory insurance paid by employees and employers. In this study we use the Finnish categorization for DPs as outcome variables: 1) Permanent full-time DP, 2) Permanent partial DP, 3) Fixed-term DP, 4) Fixed-term partial DP.

### Data collection

OH primary care data contained information on visits to different professionals (2014–2016) and were used to determine frequent attendance to OH primary care. The data were sent by Pihlajalinna to Statistics Finland, which pseudonymized the data and combined it with sociodemographic data from the FOLK-database [27]. The DP decisions were derived from Finnish Centre for Pensions (FCP), which were also sent to Statistic Finland and combined with the pseudonymized data. Tampere University processed the pseudonymized data in the information safe FIONA-environment provided by Statistics Finland.

Our initial data comprised 78,507 patients. The study material was limited to employees aged 18–68 years who had visited the OH primary care face-to-face at least once during the study years. Only illness related visits conducted face-to-face were included, and mandatory health check-ups were excluded. We excluded patients of whom there was no record in 2015 since sociodemographic data were drawn from 2015 (*n* = 445). After these exclusions our study comprised 66,386 patients.

### Statistical analysis

FAs were defined as top decile of healthcare attenders in OH primary care during 2014–2016. Details on defining FA-groups can be found described in a previous study, but a minimum of eight face-to-face visits in a year was held as a limit for becoming a FA [26]. Those patients that were in the top decile of attenders in one of the study years (2014, 2015 or 2016) were named 1-year-FA (1yFA). The patients that were in the top decile in any two study years were named 2-year-FA (2yFA). Those patients that were in the top decile in all three study years were considered persistent frequent attenders (pFA). Patients that were never in the top decile were considered as a reference group, non-frequent attenders (non-FA). The cohort used in this study is dynamic and patients could be lost to follow-up due to employment termination. A flow diagram of patient categorization and exclusions can be found in a previous paper [26].

The sociodemographic variables were derived from the Statistics Finland FOLK-database. In the descriptive part we examined occupational class divided into manual (e.g. cleaners, cooks, mechanics), lower non-manual (e.g. sales assistants, nurses), upper non-manual (e.g. managers, engineers, teachers), entrepreneur combined with farmers and lastly others [28, 29]. We also examined educational level (basic < 10 years, intermediate 10–12 years, high > 13 years) [28] and living alone [28]. All these factors were drawn from 2015 which was in the middle of our study period to describe the sociodemographic characteristics of OH primary care FAs. When examining whether sociodemographic factors predict frequent attendance to OH primary care, we used occupational class, educational level, unemployment and living alone in 2013, which was before the chosen FA-period. When examining whether frequent attendance to OH primary care was associated with disability pensions despite sociodemographic factors, we used occupational class, educational level, living alone, area of living (divided into urban, semi-urban, rural) [22], unemployment and disposable family income [28] from 2014 in adjusting the models. Matching with social determinants of health, we used occupational class to estimate employment; education for education; and living alone and area of living for social support and neighborhood factors, unemployment and disposable family income as indicators of income or poverty. The endpoint was disability pension decision (permanent full-time DP, permanent partial DP, fixed-term DP, fixed-term partial DP) in 2017–2018.

The data were analysed using R-software. In all analyses *P* values less than 0.05 were considered statistically significant. We used multinomial logistic regression with occupational class, educational level and unemployment from 2013 compared with frequent attendance to OH primary care in 2014–2016 to study if unemployment, educational level or occupational class predict frequent attendance to OH primary care. These analyses were adjusted with age and sex. In the analysis examining the predictive power of frequent attendance to OH primary care for DP decisions, the logistic regression analyses were adjusted for age, sex, occupational class, educational level, living alone, area of living, unemployment and disposable family income from 2015. We also examined if there were differences in proportions of different FA-groups being unemployed in years 2013 and 2017.

## Results

The study population after exclusions comprised 66,386 patients (2014–2016). When divided into four categories 592 (0.9%) patients were pFAs, 1602 (2.4%) 2yFAs, 6519 (9.8%) 1yFAs and 57,673 (86.9%) non-FAs.

Descriptive data are shown in Table 1. The pFAs often have an intermediate education level and belong to manual workers. Living alone also appears to be more common among FAs. Approximately 90% of pFAs were employed in 2013 and 2017, the respective proportions for non-FA were 86% in 2013 and 87% in 2017. Only 6% of pFAs and 10% of non-FAs had been unemployed for 45 days or more in 2013, while the unemployment rate in 2017 was 8% of pFAs and 10% of non-FAs (data not shown).

**Table 1:** Distribution of sociodemographic factors in different FA groups in 2015 (*N* = 66,386)

	non-FA *n* = 57,673		1yFA *n* = 6519		2yFA *n* = 1602		PFA *n* = 592	
	%	n	%	n	%	n	%	n
Male	57	32,915	50	3265	47	753	44	262
Female	43	24,758	50	3254	53	849	56	330
18–34	33	18,973	25	1609	19	307	19	111
35–44	23	13,499	24	1588	25	398	23	139
45–54	25	14,257	29	1880	30	480	32	191
55–68	19	10,944	22	1442	26	417	26	151
Occupational class								
Manual	31	17,753	41	2664	46	732	52	307
Lower non-manual	31	17,766	36	2347	36	585	33	195
Upper non-manual	19	11,014	14	918	12	190	11	63
Entrepreneurs	4	2420	2	124	1	14	< 1	–
Others*	15	8720	7	466	5	81	4	25
Educational level								
Basic	10	6040	10	681	10	157	11	65
Intermediate	49	28,364	56	3643	58	929	62	369
High	40	23,269	34	2195	32	516	27	158
Living alone								
Alone	19	10,884	20	1286	21	332	26	154
Not alone	80	46,197	80	5180	78	1256	74	436
Missing	1	592	< 1	53	1	14	< 1	–

Frequent attendance to OH primary care was defined as the top decile of attenders (frequent attender 10%, FA10)

1yFA = Patients that were in the top decile of attenders in one of the study years (2014, 2015 or 2016)

2yFA = Patients that were in the top decile in any two study years (2014, 2015 or 2016)

pFA = Patients that were in the top decile in all three study years (2014, 2015 and 2016)

non-FA = Patients that were never in the top decile were considered as a reference group, non-frequent attenders

*Unemployed, students, pensioners, others, unknown

In the two years following the FA-period, the pFA group received proportionally most DP decisions of any kind (1.9% of pFA, 1.6% of 2yFA, 1.1% of 1yFA and 0.3% of non-FA) (Table 2). The 1yFA and 2yFA groups differed only slightly so that 2yFAs had proportionally more full-time pensions than the others.

**Table 2:** Proportions (%) of disability pension decisions for different FA groups in 2017–2018

	Permanent full-time DP 2017–2018	Permanent partial DP 2017–2018			Fixed-term DP 2017–2018		Fixed-term partial DP 2017–2018	
	%	n	%	n	%	n	%	n
non-FA (n = 57,673)	0.3	158	0.3	180	0.2	121	0.1	38
1yFA (n = 6519)	1.1	69	1.0	64	1.0	64	0.4	25
2yFA (n = 1602)	1.6	26	1.1	19	1.6	26	0.3	5
pFA (n = 592)	1.9	11	1.5	9	1.9	11	0.8	5

Frequent attendance to OH primary care was defined as the top decile of attenders (frequent attender 10%, FA10)

1yFA = Patients that were in the top decile of attenders in one of the study years (2014, 2015 or 2016)

2yFA = Patients that were in the top decile in any two study years (2014, 2015 or 2016)

pFA = Patients that were in the top decile in all three study years (2014, 2015 and 2016)

non-FA = Patients that were never in the top decile were considered as a reference group, non-frequent attenders

Being a manual worker in 2013 was associated with frequent attendance to OH primary care in the following years for both genders, when compared to upper non-manual workers (Table 3). Specifically, lower non-manual work was associated with being 1yFA- and 2yFA for both genders but with pFA for only females. Concurrently, basic and intermediate education were associated with frequent attendance to OH primary care for males, but for females only intermediate education was significant. Entrepreneurship on the other hand was associated with a lower likelihood of being FA for both genders. Being unemployed in 2013 was not linked to frequent attendance to OH primary care in the following years but, on the contrary, it was linked to a lower likelihood to being in the 2yFA in females but not in males. Living alone was linked with being pFA for both sexes.

**Table 3:** Sociodemographic factors in 2013 associated with frequent attendance to OH primary care in 2014–2016 in multinomial logistic regression (adjusted for age)

	1yFA		2yFA		pFA	
	Female	Male	Female	Male	Female	Male
	OR (95% CI)					
Occupational class*						
Manual	1.58 (1.39–1.79)	1.92 (1.73–2.14)	2.37 (1.86–3.01)	2.85 (2.24–3.62)	3.02 (2.05–4.44)	2.93 (1.97–4.34)
Lower non-manual	1.41 (1.27–1.58)	1.14 (1.00–1.30)	1.67 (1.34–2.09)	1.52 (1.14–2.03)	1.71 (1.19–2.47)	0.98 (0.58–1.65)
Entrepreneur	0.5 (0.36–0.69)	0.73 (0.59–0.92)	0.25 (0.10–0.62)	0.39 (0.20–0.76)	0.28 (0.07–1.16)	0.22 (0.05–0.92)
Others	1.08 (0.93–1.27)	1.10 (0.94–1.28)	0.77 (0.54–1.11)	1.20 (0.85–1.71)	1.03 (0.58–1.82)	1.06 (0.59–1.91)
Education**						
Basic	1.06 (0.92–1.21)	1.58 (1.40–1.78)	0.87 (0.65–1.15)	1.52 (1.18–1.95)	1.18 (0.77–1.83)	2.02 (1.29–3.16)
Intermediate	1.34 (1.24–1.45)	1.76 (1.62–1.93)	1.53 (1.33–1.77)	2.00 (1.67–2.40)	2.07 (1.63–2.62)	2.79 (1.99–3.90)
Unemployment***	0.97 (0.87–1.08)	0.95 (0.86–1.06)	0.65 (0.51–0.83)	0.91 (0.73–1.13)	0.72 (0.49–1.05)	0.73 (0.49–1.08)
Living alone	1.07 (0.97–1.18)	1.03 (0.94–1.13)	1.15 (0.96–1.37)	1.12 (0.94–1.35)	1.35 (1.02–1.77)	1.54 (1.17–2.04)

Frequent attendance to OH primary care was defined as the top decile of attenders (frequent attender 10%, FA10)

1yFA = Patients that were in the top decile of attenders in one of the study years (2014, 2015 or 2016)

2yFA = Patients that were in the top decile in any two study years (2014, 2015 or 2016)

pFA = Patients that were in the top decile in all three study years (2014, 2015 and 2016)

non-FA = Patients that were never in the top decile were considered as a reference group, non-frequent attenders

OR = odds ratio

CI = confidence interval

* = reference upper non-manual, ** = reference high education, *** = reference no unemployment in 2013

Confidence intervals (CI) estimated under normal distribution assumption

When studying the individual predictive power of frequent attendance to OH primary care being FA was predictive of permanent full-time DP and any DP, including also short-term solutions for both sexes (Table 4). This association grew stronger as frequent attendance to OH primary care persisted for two or more years. The associations differed slightly for male and female with 2yFAs, the association being stronger with females (OR for permanent full-time DP 7.96 (4.54–13.95) for females and 2.31 (1.10–4.83) for males. Frequent attendance to OH primary care was predictive of DP even when adjusted for confounding socioeconomic factors.

**Table 4:** Predictive value of frequent attendance to OH primary care in 2014–2016 for disability pension in 2017–2018 when adjusted for age, sex, occupational class, educational level, unemployment, living alone, income and area of living

	OR (95% CI)	
	Female	Male
Permanent full-time DP		
1yFA	2.9 (1.77–4.75)	4.01 (2.78–5.78)
2yFA	7.96 (4.54–13.95)	2.31 (1.10–4.83)
pFA	4.08 (1.44–11.58)	5.81 (2.59–13.05)
Any DP		
1yFA	3.29 (2.59–4.17)	3.66 (2.90–4.6)
2yFA	6.19 (4.52–8.49)	2.48 (1.56–3.92)
pFA	5.07 (3.09–8.33)	5.76 (3.36–9.89)

Frequent attendance to OH primary care was defined as the top decile of attenders (frequent attender 10%, FA10)

1yFA = Patients that were in the top decile of attenders in one of the study years (2014, 2015 or 2016)

2yFA = Patients that were in the top decile in any two study years (2014, 2015 or 2016)

pFA = Patients that were in the top decile in all three study years (2014, 2015 and 2016)

non-FA = Patients that were never in the top decile were considered as a reference group, non-frequent attenders

OR = odds ratio

CI = confidence interval

## Discussion

This study provides new understanding on frequent attendance to OH primary care and its associations with work disability. The finding that frequent attendance to OH primary care is associated with work disability despite sociodemographic factors, is novel. This is also the first study to describe the sociodemographic factors of FAs in OH primary care, representing the working population in Finland.

For the first time, in this study, we were also able to analyse the sociodemographic factors associated with frequent attendance to OH primary care in OH primary care. The finding that manual and lower non-manual workers were overrepresented in the FA group was expected. Social determinants of health, including employment conditions, gender, income, and education influence health status [30]. The relationship between the social determinants of health and health outcomes including morbidity and mortality is complex. In general, it could be thought that as a person lives in a context with poor social determinants of health, possibly being poorer, of lower education, discriminated against and living in an unhealthy neighbourhood and generally having fewer opportunities in life, morbidity and mortality rates increase and thus visits in occupational health services may increase. However, in this study frequent attendance to OH primary care was predictive of DP even when adjusted for confounding socioeconomic factors. This may be due to this study population consisting of employed persons, who may already be a healthier population than those who are not active in the workforce. However, the effect of social determinants of health could be seen in the gender differences in frequent attendance in the study, with women more often being frequent attenders to OH primary care than men. Whether this is due to different conditions, or different work characteristics, needs further study. Furthermore, it should be noted that the social and health inequities in Finland are among the lowest in Europe [31].

Having less vocational education and lack of professional education have been linked to frequent attendance to OH primary in GP setting [32, 33] and also to DP [18, 19]. This finding was verified in this setting, with data consisting of the working population alone. Interestingly, intermediate education appeared to have a stronger association with frequent attendance to OH primary than basic education did. This could be associated with psychological and physical demands of their work, however this finding requires further examination. We have previously perceived an association with human health and social work activities, that could be linked educational level also [4].

Entrepreneurs and upper non-manual workers are less likely to be FA. Entrepreneurs pay for their OHS themselves, which might affect their service use. Entrepreneurs are entitled to compensation from the state for OHS, but nevertheless, only a minority of sole entrepreneurs organise OHS for themselves [34]. Entrepreneurs are also underrepresented in our data compared to the general population [35], which might affect the perceived association.

Non-manual work is less physically strenuous than manual work and the physical demands are lower. Thus, the previously perceived association with musculoskeletal disorders of the FAs [36] may be explained by their occupational class and the demands of their work. On the other hand, physically demanding working conditions and manual work are also linked to DPs [37]. A previous study found an association between frequent attendance to OH primary care and manufacturing industry and human health and social work activities [4]. That study could not control for the impact of occupational class, a gap that this study addresses. Manual and lower non-manual work is common in these industries, that are also often physically demanding. In our study lower manual work in particular was associated with FA, therefore it appears that patients working in manual professions might benefit from planned healthcare services when an illness affecting work ability arises.

In the GP setting, frequent attendance to OH primary care has been linked to unemployment [5, 9] and unemployment is also linked to DP risk [38]. In this setting of OH primary care, being unemployed in 2013 was not associated with frequent attendance to OH primary care in the following years. There might be several reasons behind this. It is possible that one might enter working life only briefly after unemployment periods and thus might not “have time” to become FA. On the other hand, one might underuse services or not use services provided by the employer in the fear of losing work. Interestingly, the data indicate that over the years some pFAs drifted towards longer unemployment periods. This was not visible among the non-FAs. Studies suggest that sickness absences are a risk for unemployment and job termination for temporary jobs [39] and this could be one explanation. Future studies are needed to study the patients that are lost to lollow-up in the OHS.

A previous study showed that frequent attendance is linked to work disability in the near future [17]. Previous studies have also shown a link with prolonged sickness absences [16, 40]. Also, low socioeconomic status has been linked to sickness absences for mental disorders in frequent users of OHS [15]. The current study examined disability pensions in the years following frequent attendance, taking into account the potential confounding effects of sociodemographic variables. These analyses suggest that frequent attendance is associated not only with permanent full-time DP but also any DP, despite the underlying sociodemographic factors (age, sex, occupational class, educational level, unemployment, living alone, disposable family income and area of living). The sociodemographic factors are rarely available in any medical records – thus they cannot be used in identifying disability risks although they are known the be linked to increased risk for work disability. Frequency of visits is, however, easily available through medical records and can be used to identify persons at risk of work disability risk. The results can be easily taken into practice and thus aid health service providers and physicians. This finding emphasizes the value of consultation frequency as an additional indicator for identifying disability risks.

It should be noted, that other DP decisions than permanent full-time DP (i.e. fixed-term and partial solutions) are endpoints that reflect the realization of work disability but at the same time they are also supportive measures to help patients stay and return to working life. Fixed-term DP decisions are meant for the period necessary for recovery and only approximately half lead to permanent DP during a four-year follow-up [41]. Partial DPs allow for staying in the workforce and are particularly useful towards the end of the working career, when some work ability remains despite illnesses and disabilities. This often requires work modifications in the workplace which are agreed upon during OH negotiations [42], which are a supportive structure of the Finnish OHS. Identifying patients with work disability risks in all healthcare sectors and steering them to OHS to initiate support mechanisms at the workplace and in OHS is crucial.

OHS in Finland aim to prolong working careers and help maintain work ability. Sickness absence monitoring is most commonly used in identifying individuals at risk of work disability [43, 44]. This is, however, a rather late indicator and additional possibly earlier indicators would be welcome. Identifying persons at risk of DP who need additional support could work best through using several indicators. Currently persons with lowered work ability and risk of DP are identified at the physicians’ office [45] and through a self-evaluation questionnaire [46]. Once identified, the multifaceted support mechanisms of OHS should be exploited to find solutions that restore work ability: OH negotiations to agree on necessary work modifications, educational training or vocational rehabilitation are just some measures that aim to prolong staying in the workforce. Instituting these measures earlier could support protecting work ability and co-operation between healthcare sectors.

This study provides new understanding of frequent attenders of OH primary care and a unique view on the working population. The studied population is large and represents the working population in Finland quite well, with patients from both rural and urban areas. There are slightly fewer municipal workers than in the general population and thus the manufacturing industry is accentuated. The study design, combining routine medical record data that are easily available with large register data is an emerging field of study providing important information. Registers in Finland are generally of good quality and have minimal numbers of missing data. The follow-up study design allows for a more complete understanding of the associations between sociodemographic factors and service use. Although OH primary care as such does not exist outside Finland, our results allow some generalisation to the working population also outside Finland, often treated by GPs. Patients that had no visits to OH primary care during the years were not included, since their data are not available in the medical record database. Despite the large cohort, the group of pFAs represents less than 1% of the whole study population (however containing 592 patients), which might lead to lacking statistical significance. It should also be noted, that patients that fall ill very rapidly, might end up on DP without frequent visits to OH primary care. Illnesses such as serious cancers and heart failures, could be an example. Due to the dynamic nature of the cohort, patients may be lost to follow-up if their employment ends. This is a limitation, but the large sample dilutes the effect. Furthermore,this study is limited by the inability to track the use of other healthcare services over the years. This should be studied in the future, as unemployment might steer service use to other sectors. Whether this affects disability risks should be examined.

## Conclusions

Frequent attendance to OH primary care is associated with disability pensions despite underlying sociodemographic differences. These individuals should be identified by professionals treating working patients and rehabilitative needs and other measures necessary to help staying in the working life should be used early on. Consultation frequency is an additional measure that could be used alongside other measures in identifying patients in need of additional support.

## Data Availability

The data supporting the findings are combined for the purposes of this study and are stored by Statistics Finland. Restrictions apply to the availability of these data, which were used under license for the current study, and so they are not publicly available.

## Abbreviations

CI: Confidence interval
DP: Disability pension
FA: Frequent attender
FCP: Finnish Centre for Pensions
GP: General practice
OH: Occupational health
OHS: Occupational health services
OR: Odds ratios
pFA: Persistent frequent attender
